# Answer to the editor: “Factors influencing the quality of life of children with cochlear implants”

**DOI:** 10.1016/j.bjorl.2020.09.001

**Published:** 2020-09-29

**Authors:** Vagner Antonio Rodrigues da Silva, Alexandre Caixeta Guimarães, Arthur Menino Castilho

**Affiliations:** Universidade Estadual de Campinas (Unicamp), Faculdade de Medicina Ciências (FCM) Departamento de Otorrinolaringologia, Cirurgia de Cabeça e Pescoço, Campinas, SP, Brazil

Dear Editor,

We read with interest the article that was recently published by Silva JM et al., “Factors influencing the quality of life of children with cochlear implants”.^1^ The authors’ objective was “to evaluate the factors influencing the quality of life of children with cochlear implants (CI), considering the age at surgery, the hearing age, the age at assessment, the hearing skills, the spoken language, the degree of family permeability, and the schooling and socioeconomic status of the parents”.

We congratulate the authors for their invaluable work, but we would like to ask some questions:

1. What do you consider as socioeconomic class: lower low, upper low, lower medium, medium, upper medium class?

2. Why did you assess children with unilateral CI in the study?

3. Regarding the causes of hearing loss, what is the “family history” considered? Genetics?

4. Questionnaires used:

"Family Involvement Rating"

“Children with cochlear implants: parent's perspectives (CCIPP)”

“The quantitative responses were analyzed using the software parent questionnaire manager – parent views and experiences questionnaire data entry software (ParQ120.exe., version 1.02: ISVR software, copyright 2003)”

You used these three questionnaires translated into Portuguese, but have they been validated?

5. In the authors’ conclusion, we observed some important points: “Conclusion: The influencing factors that correlated with the quality of life of the implanted children were the older age at evaluation, better hearing and language skills, the mother's level of schooling and the family’s permeability”.

5.1 “The older age at the evaluation” - Does it imply a longer time of implant use and speech therapy rehabilitation and, thus, would they have a better quality of life? What are the explanations the authors attribute to this difference in quality of life according to age at the evaluation, since this information is quite conflicting with the literature as mentioned in the discussion. Regarding the age at surgical procedure, we found several articles in the literature that show an association between younger age at implantation and better hearing results.^2^, ^3^, ^4^ It could be expected that children implanted earlier would have better quality of life results as well. With a larger sample, perhaps this association could be observed. Possibly, the fact that this study only included implanted children up to a maximum age of 3 years and 6 months reduced the difference in the results, since children implanted after 3 years of age have worse hearing results than those implanted earlier.

5.2 “Better auditory and language skills” – they depend on a number of factors: cause of deafness, time of the implant daily use, adequate speech therapy, family commitment, living with other children and school.^4^

5.3 The second paragraph of the conclusion “This knowledge can guide the rehabilitation speech therapist to promote improvements in the planning of specialized speech therapy…”; we consider that although the points presented herein are important, they should not be part of the conclusion, but of the discussion of the article.

## Conflicts of interest

The authors declare no conflicts of interest.
